# The telomeric part of the human chromosome 21 from *Cstb* to *Prmt2* is not necessary for the locomotor and short-term memory deficits observed in the Tc1 mouse model of Down syndrome

**DOI:** 10.1016/j.bbr.2010.10.023

**Published:** 2011-03-01

**Authors:** Arnaud Duchon, Stéphanie Pothion, Véronique Brault, Andrew J. Sharp, Victor L.J. Tybulewicz, Elizabeth M.C. Fisher, Yann Herault

**Affiliations:** a Institut de Génétique Biologie Moléculaire et Cellulaire, Translational Medicine and Neuroscience Program, IGBMC, CNRS, INSERM, Université de Strasbourg, UMR7104, UMR964, 1 rue Laurent Fries, 67404 Illkirch, France; b Transgenese et Archivage Animaux Modèles, TAAM, CNRS, UPS44, 3B rue de la Férollerie 45071 Orléans, France; c Department of Genetics and Genomic Sciences, Mount Sinai School of Medicine, 1425 Madison Avenue, Room 14-75B, Box 1498, New York, NY 10029, USA; d MRC National Institute for Medical Research, Mill Hill, London NW7 1AA, UK; e UCL Institute of Neurology, Queen Square, London WC1N 3BG, UK; f Institut Clinique de la Souris, ICS, 1 rue Laurent Fries, 67404 Illkirch, France

**Keywords:** Mouse model, Aneuploidy, Mental retardation

## Abstract

Trisomy 21 or Down syndrome (DS) is the most common form of human aneuploid disorder. Increase in the copy number of human chromosome 21 genes leads to several alterations including mental retardation, heart and skeletal dysmorphologies with additional physiological defects. To better understand the genotype and phenotype relationships, several mouse models have been developed, including the transchromosomic Tc1 mouse, which carries an almost complete human chromosome 21, that displays several locomotor and cognitive alterations related to DS. In this report we explore the contribution of the genetic dosage of 47 mouse genes located in the most telomeric part of Hsa21, using a novel model, named Ms4Yah, carrying a deletion of the 2.2Mb *Ctsb*–*Prmt2* genetic interval. We combine this deletion with the Tc1 Hsa21 in a rescue experiment. We could recapitulate most of the Tc1 phenotypes but we found no phenotypes induced by the Ms4Yah and no contribution to the Tc1-induced phenotypes even if we described new alteration in social preference but not in olfaction. Thus we conclude that the genes conserved between mouse and human, found in the most telomeric part of Hsa21, and trisomic in Tc1, are not contributing to the major Tc1 phenotypes, suggesting that the *Cstb*–*Prmt2* region is not playing a major role in locomotor and cognitive deficits found in DS.

## Research highlights

▶ Locomotor deficits of the Tc1 DS model are not rescued by the Ms4Yah deletion of the *Cstb*–*Prmt2* region. ▶ Tc1 and Tc1/Ms4Yah mice show specific exploratory behaviour in the open field. ▶ Memory impairment in Tc1 mice is not affected by the deletion of the *Cstb*–*Prmt2* region. ▶ Tc1 and Tc1/Ms4Yah mice exhibit impaired social preference. ▶ Olfaction is not impaired in the Tc1 mouse model.

## 1 Introduction

Down syndrome (DS) or trisomy 21 is the most common genetic form of mental retardation. This anomaly of chromosome number affects one out of 700 births with a sex ratio of three boys for two girls [21]. An epidemiological study has estimated the world-wide incidence of the syndrome to be more than 20,000 cases per year. However, these numbers are probably under estimated if we take into account that DS represents around 2% of the spontaneous abortions [8]. A recent study carried on the United Kingdom population shows that antenatal diagnosis has reduced the birth of DS children by only about 1% in 20 years, while the number of detected cases has increased by 71% during the same period. Absence of screening would therefore have resulted in an increase of 48%, principally due to increased maternal age, which is the main risk factor [35].

Two aspects of the disease are particularly disabling for the patients beside the DS cardiovascular alterations which are now treated and cured in most of the cases, the mental retardation (MR) and, to a lesser extent, the locomotor deficits that are widespread in DS patients [50]. DS individuals are often described as clumsy and have deficit in motor skills [46]. In particular, patients show difficulties in planning and achieving movements [33], leading to grasping difficulties [24]. Mental deficit is found in all DS patients, with an average intelligence quotient (IQ) of 50 [49]. Only one case of partial trisomy without MR has been described in the literature [48]. MR is the result of deficits in several types of memories. Short-term verbal memory is particularly impaired, certainly due to a limited capacity of the verbal short-term memory system [51,44,3] that could be at the origin of the impaired language seen in DS patients [44,28,30].

To a lesser extent, visuo-spatial working memory is also affected [52]. It was shown that this deficit is more pronounced when patients have to remember simultaneously visualized patterns of localisation compared to more simple exercises of sequential memorization [28]. In these two systems it clearly appears that the degree of deficit is correlated to the degree of participation of the “central administrator” [29,30]. While children display intact implicit memory, exercises requiring explicit memory indicate that this type of memory is deeply affected. This difference is probably due to difference between the two systems. Indeed, the first one works with automated processes that require little attention whereas the second requires more conscious attention and needs a strategy for coding and retrieving the information. Hence, DS patient studies point at a deficit in the capacity of coding and retrieving the information. But the exact nature of those deficits is still unclear.

DS does not affect the different structures of the brain equally. MRI studies have shown that medial parts of the temporal lobe and the hippocampus are mostly affected. In these structures, the number of neurons and the length and number of the dendritic connections are greatly reduced (for review see [8]). In addition, a neuropsychological cognitive approach on DS adolescents using tests that were first developed in animal models indicates hippocampal dysfunction similar to those observed in one mouse DS model [42].

Indeed, the mouse shares many homologies with humans at the cellular, biochemical and molecular level. In addition, behaviour such as anxiety, stress, memory, emotional responses and locomotor activities are well modelized in this organism. At the genomic level, the long arm of the human chromosome 21 (Hsa21) is approximately 33.7 Mb in length and contains about 430 protein coding genes of which 293 have a homolog in the mouse genome. These genes are found in syntenic regions localised on three different mouse chromosomes. From the centromeric to the telomeric end of the Hsa21, the first and largest region is found on the Mmu16 (about 37 Mb in length and 224 orthologs between the *Lipi* and *Zfp195* genes), followed by a smaller region on Mmu17 (1.1 Mb in length and 22 genes between *Umodl1* and *Hsf2* bp) and finally a region on Mmu10 (2.3 Mb in length with 47 genes between *Cstb* and *Prmt2*). In these three syntenic regions, relative order and orientation of the genes are preserved between the two species (for review [13]).

Most of the existing mouse models for DS are trisomic for parts of the Mmu16 homologous region, corresponding to the centromeric region of Hsa21. Few investigations have been carried out so far on genes present on the most distal part of Hsa21. Only two recent mouse models have been generated that comprise genes from the telomeric region: the Tc1 transchromosomic mouse model containing an almost complete (∼82%) human chromosome 21 [38] and a model trisomic for all human chromosome 21 syntenic regions [56].

We decided to investigate the contribution of the *Cstb*–*Prmt2* genetic interval to the DS phenotype. Indeed this region encompasses 47 genes of which 12 have been shown to induce loss-of-function phenotypes in various tissues and affect several functions including homeostasis and behaviour (derived from MGI phenotypic database). Thus we combined a deletion of the *Cstb*–*Prmt2* genetic interval carried in our Ms4Yah mouse model monosomic with the Hsa21 present inTc1 model. The Tc1 model displays phenotypes associated with DS that are due to the trisomy of 92% of Hsa21 genes [38]; 1 human copy and two mouse copies), in particular defective working memory and characteristic locomotor deficits have been clearly documented [34,19]. The relevance of such a rescuing approach has already been demonstrated in other studies in order to decipher the role of the Down syndrome Critical Region in the appearance of the craniofacial phenotype in another DS mouse model, the Ts65Dn mouse [39], or the contribution of single gene [5]. Thus, mating Tc1 with Ms4Yah mice will reduce the trisomy of about 47 genes located in the *Cstb*–*Prmt2* interval, and the subsequent characterization of phenotypes in Tc1/Ms4Yah mice should help us to determine the contribution of the genes present in the telomeric part of the Hsa21 to the deficits present in Tc1 mice, by bringing back to two copies the genes present within the *Cstb*–*Prmt2* interval. In parallel we also studied the consequences of the deletion of the 47 genes located from the *Cstb*–*Prmt2* region in the Ms4Yah mice. This analysis complete the analysis for the smaller interval from *Col6a1* to *Prmt2* studied previously as a model of Monosomy 21 [4] and enables us to better understand the role of the telomeric region of Hsa21 and to decipher the mechanisms leading to cognitive deficits in DS.

## 2 Materials and methods

### 2.1 Engineering new mouse lines

Mice bearing the deletion of the chromosomal region between the *Cstb* and *Prmt2* genes were obtained by Cre-loxP recombination in an in vivo approach [14]. Briefly, loxP sites were introduced at the *Cstb* and *Prmt2* loci in the same relative orientation in the mouse genome using two successive rounds of homologous recombination in ES cells. Mice containing the two loxP sites were mated with the mouse line TgN(Zp3-Cre)Knw, expressing the Cre recombinase under the control of the Zona pellucida 3 gene promoter active in oocytes [12]. Females born from this mating and bearing both the ZP3Cre transgenes and loxP sites on the same chromosome (cis configuration) are mated with wild-type C57BL6/J males. In oocytes of these females, Cre expression induces loxP site recombination leading to the deletion of the *Cstb*–*Prmt2* region in female gametes and of offspring monosomic for this region. Ms4Yah mice were obtained with a frequency of 3 animals out of 83 born.

The Tc1 transchromosomic line was created by Fisher and Tybulewicz, and has been described previously [38]. All the lines were kept on an F1 B6C3B background; the C3B are sighted C3H/HeH, a congenic line for the BALB/c allele at the *Pde6b* gene [20].

### 2.2 Maintenance and breeding of the mice

The mice were bred under SPF conditions and were treated in compliance with animal welfare policies from the French Ministry of Agriculture (law 87 848 and YH accreditation 45–31). Tc1 animals cannot be kept on a pure genetic background due to the loss of transmission of the Hsa21 [38]. In this study, the Tc1 line was maintained by crossing Tc1 females with F1B6C3B males (see Section 5). The Ms4Yah line was generated on 129P2/OlaHsd background and backcrossed for 9 generation on C57BL/6J (B6). Before mating the Ms4Yah mice with Tc1, females Ms4Yah were first mated with C3B males to produce F1 B6C3B Ms4Yah males to mate with Tc1 F1B6C3B females to produce a cohort of 14 Ms4Yah, 14 Tc1, 14 Tc1Ms4Yah and 13 wild-type males for behavioural experiments. A second group of 18 Ms4Yah, 13 Tc1, 12 Tc1Ms4Yah and 18 wild-type controls was produced to confirm some results.

### 2.3 Mouse genotyping

For both the identification of the Ms4Yah allele and the Tc1 transgene, genomic DNA was isolated from tail biopsies using the NaCl precipitation technique. The Ms4Yah allele was identified using Southern blot analysis. About 10 μg of DNA was digested with a specific restriction enzyme. DNA fragments were separated on a 0.7% agarose gel and transferred on a nylon membrane. The membrane was then hybridized with a specific DIG-labelling probe that recognizes the ampicilin gene present on the insertion vector and remaining in the Ms4Yah allele and revealed with CDP Star (Roche).

The Hsa21 present in Tc1 mice was identified by PCR using primers D21S55F (5′-GGTTTGAGGGAACACAAAGCTTAACTCCCA-3′) and D21S55R (5′-ACAGAGCTACAGCCTCTGACACTATGAACT-3′) that are specific for the Hsa21 and control primers specific for the mouse genome (MyoF: 5′-TTACGTCCATCGTGGACAGCAT-3′, MyoR: 5′-TGGGCTGGGTGTTAGTCTTAT-3′) giving PCR products of 208 bp and 245 bp respectively.

## 3 Experimental procedures

### 3.1 Array-based comparative genomic hybridization

To confirm the deletion of the *Cstb*–*Prmt2* region in the Ms4Yah model, a gene copy number variation (CNV) study on the whole genome was undertaken using Nimblegen mouse HD2 whole genome CGH oligonucleotide arrays. Comparative analysis was done using DNA extracts from one animal bearing the *Cstb*–*Prmt2* deletion and from one wild-type animal that were fluorescently labelled with Cy5 (wildtype) and Cy3 (Ms4Yah). After sonication and labelling, DNA is hybridized to the CGH array, followed by washing of the slide according to manufacturer's instructions (Roche Nimblegen, Madison, WI, USA). Slides were scanned using a G2565 scanner at 3 μm resolution (Agilent Technologies, Palo Alto, CA, USA), and array images analysed using NimbleScan v2.5 software (Roche NimbleGen, Madison, WI, USA) with default parameters incorporating spatial correction. Arrays comprised 2,100,000 isothermal probes 50–75 bp length, with a median spacing of 1.1 kb throughout the genome (UCSC NCBI37/mm9/July 2007 assembly), enabling high-resolution CNV detection between Ms4Yah and the wild-type control. A similar experiment was conducted on Tc1 mice to check the integrity of the Hsa21 (Fisher and Tybulewicz, personal communication).

### 3.2 Behavioral analysis

A series of behavioral experiments were conducted in order to evaluate cognition and motor conditions in the mice. For all these tests, mice were kept in SPF conditions with free access to food and water. The light cycle was controlled as 12 h light and 12 h dark (lights on at 7 AM). The MWM was conducted between 9:00 AM and 1:00 PM. All the over tests were done between 9:00 AM and 4:00 PM. The different apparatus used in this study was placed in a dimly lit testing room (18–20 lux). To produce experimental groups, only animals coming from litters containing a minimum of two male pups were selected. After weaning, male mice were gathered by litters in the same cage. Animals were transferred to the experimental room 30 min before each experimental test. The tests were administered in the following order: Morris water maze (working memory, at age 4.5 months), rotarod and Lipp bar-crossing tests (locomotor, at age 7 months), open field (at age 8 months) and sociability test (at age 9 months). The open field and sociability experiments were duplicated with a second independent younger group of animals (at age 6 months and 7 months respectively, data not shown). Animals were also tested for their olfactory sensitivity. One week was kept between two behaviours tests.

#### 3.2.1 Rotarod

The purpose of the Rotarod test is to assess rodent's sensorimotor coordination. The evaluation criterion is the time that the mouse spends on the rotating rod before falling. The apparatus (Bioseb, France) is made of a rotating bar of 5 cm diameter (hard plastic materiel covered by grey rubber foam) on which mice are placed facing the direction of rotation. The procedure was adapted from Galante et al. [19]. The animals are first habituated to stay onto the rod for 30 s at a constant speed of 4 rotations per minute (rpm). This is followed by 3 days of training with 4 trials per day with about 1 h between each trial. During these trials, mice are placed on an accelerating rod increasing from 4 rpm to 40 rpm in 5 min. The test is stopped when the mouse fall from the rod or when there is more than one passive rotation. The latency to fall and the maximal speed before falling are recorded. On the fourth day, mice have two test sessions. They consist of 6 consecutive trials of 2 min at constant speed, with increasing speed between each trial (4, 10, 16, 22, 28, 34 and 40 rpm). For each trial, the elapsed time until the mouse fell off the rod was recorded.

#### 3.2.2 Notched bar test

This protocol is to test hindlimb motor coordination [53]. Mice are first trained on a “training” bar consisting of a natural wooden piece 1.7 mm large and 50 cm long bearing terminal platforms of 6 cm × 6 cm. Once they have successfully crossed the bar 10 times, they are tested on a special notched bar having 12 intermediate raised step equally distributed between the two platforms. Again, mice have to cross the bar 10 times, but this time, two observers are placed symmetrically to the bar, looking for hind-paw slips outside of the steps and scoring them as errors. Data are given as percentage of errors on total number of crossed steps. The two sessions (trained and test) were done the same day, with intersessions time of 4 h.

#### 3.2.3 Open field

This test measures rodent behavioral responses such as locomotor activity, hyperactivity and exploratory behaviours within a closed space. The test was carried out in a 55 cm diameter round box and mouse activity was recorded with a video tracking system (Ethovision, Noldus, France) during a unique session of 30 min. After each mouse trial, the arena was thoroughly cleaned to minimize olfactory cues. The arena was cleaned with water and dried with paper towels between each trial.

#### 3.2.4 Morris water maze working memory

This version tests short-term learning. Our protocol was adapted from the already established protocol [23,34]. In this version, the hidden platform position was changed pseudo-randomly every day during 11 days. The sequence of positioning for the platform was as followed: SE, NE, NW, SW, SE, SW, NW, NE, SW, NE, SE. Each day, mice had one training session consisting of four consecutive 60 s trials with inter-trial interval of 20 s and with the platform at the same position for all the four trials but with starting positions changing between each trial with departures from each cardinal point [north, south, east, west (N, S, E, W)]. In order to minimize the use of non-spatial strategies such as the learning of a series of movements to reach the platform, special care was taken for the choice of the platform positioning versus the starting position during the course of the experiment. Upon finding of the platform or after 60 s without finding the platform, mice were left 20 s on the platform. After the 4 trials, a fifth trial is undertaken allowing the mouse to search for the platform for 30 s. During the fifth trial of days 1, 4 and 10, the platform is removed to assess spatial memory (probe test). Travelled distances were analysed for trials 1–4 and the probe tests. An annulus crossing index (ACI) is calculated to represent the number of times the animal crosses the platform position in the quadrant that contained the escape platform minus the number of times that it crosses over platform sites in the three other quadrants. This index allows us to differentiate spatial memory from non-spatial search strategies. Mice adopting a circular swimming strategy at a constant distance from the wall will have a crossing index close to zero, mice looking for the platform at the wrong place will have a negative index, while mice having a good spatial strategy will have a positive index. Classification of search strategies was done for the session starting only on days 6–11 because, owing to the difficulty of the task (mice have to learn that the platform is always in the swimming pool but at different positions), it took time for the mice to set up their strategy [23]. To go further into the analysis, search strategies were classified into six categories in a blind manner (the experimenter did not know the genotype of the animals before the classification): thigmotaxy characterized by persistent swim along the wall; random searching with swimming in the entire pool; circling with swimming in tight circles; scanning consisting of a search in the centre of the arena; chaining with swimming in large circle at constant distance from the wall corresponding to the position of the platform; focal search characterized by taking a precise direction and searching within a precise location: this can be associated with the correct target (focal target) or a wrong position (non focal target) [23].

*Cued-platform version*: In this version, the platform is made visible to the mice by a small dark ball placed 12 cm on top of the platform, while the external cues are hidden by surrounding the pool with a black curtain. In order to be sure that the mouse uses the platform cue, the position of the platform is changed in each trial. The experiment consists of four consecutive trials.

#### 3.2.5 Three-chambered social behavior test

The system, available from Stoelting (Dublin, Ireland), is composed of three successive identical chambers (20 cm × 40 × cm 22(height) cm) with 5 cm × 8 cm openings allowing access between the chambers. The protocol is similar to the one described by Moy and collaborators [36,37]. During the habituation phase, the test mouse is placed in the middle chamber and allowed to explore the three chambers under video tracking for 10 min, with each of the two side chambers containing an empty wire cage. The second phase of the test corresponding to the sociability test is carried out directly after the habituation phase. The test mouse is enclosed in the central box, while an unfamiliar mouse (stranger 1) is placed in one of the wire cages in a random manner. The doors are re-opened and the test mouse is allowed to explore the entire social test box for 10 min. Time spent in each of the chambers, the number of entries into each chamber and the time spent sniffing each wire cage are recorded. The third phase tests the preference for social novelty. A new stranger mouse (stranger 2) is placed into the empty wire cage and the test mouse is allowed to explore again the entire social test box for 6 min, having the choice between the first, already-investigated mouse (stranger 1) and the novel unfamiliar mouse (stranger 2; Fig. 5A). The same measures are taken as for the sociability test. The entire social test box is washed under tap water and dried with absorbent paper between each test mouse to remove odours and the time spent in each lateral chamber during the habituation phase is recorded to check for place preference.

Stranger mice are adult C57BL/6J males (age 3–4 months) that were maintained in a different room from the test mice to avoid olfactory and visual contacts. Several days before the test, stranger mice are habituated to the test in the wire cage 5–10 min per day for 5 days. Each stranger mouse is only used once per test day and chosen randomly for either the sociability session or the preference for social novelty session.

#### 3.2.6 Olfactory test

This test is undertaken in order to validate the sociability test based principally on olfaction. This test is adapted from the one described by Ferguson et al. [18]. Mice are placed 5 times 2 min in a small arena (22 cm × 15 cm) containing a perforated tube (*H*: 4 cm, diameter: 3 cm) in which lies a small piece of Whatman paper soaked with orange flower water. Inter-trial time is 8 min. This is followed by a sixth trial of 2 min during which the orange flower extract is replaced by vanilla extract and the time spent sniffing the new odour is recorded.

### 3.3 Statistical analyses

ANOVA was performed to analyse differences between the 4 genotype groups using the SIGMA STAT^®^ systat Software. Post hoc analysis was done using Tukey's method. Data are presented as mean ± S.E.M.

## 4 Results

### 4.1 The Ms4Yah allele and Tc1 transchromosome are transmitted at a Mendelian ratio in the F1B6C3B genetic background

**Fig. 1:**
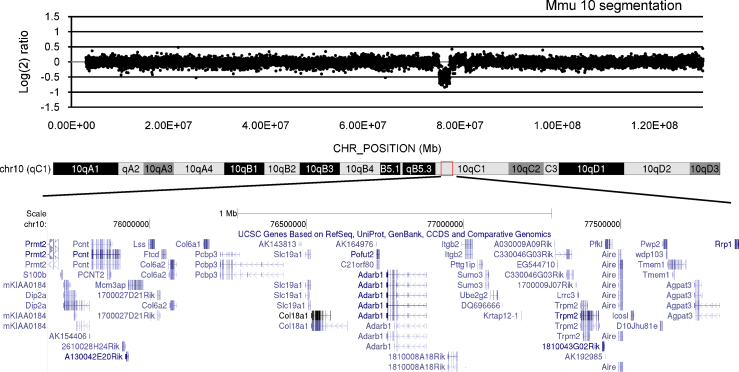
CGH profile of Mmu10 in Ms4Yah mice confirmed the loss of one copy of the genes contained within the *Cstb*–*Prmt2* interval. Plotted are log_2_ transformed hybridization ratios of Ms4Yah mouse versus wild-type mouse DNA. In the bottom of the figure is indicated the localisation of the genes inside the interval.

Ms4Yah mice are heterozygous for the deletion of the *Prmt2*–*Cstb* region that was generated *in vivo* by chromosomal engineering [14]. This 2.3 Mb region on Mmu10 is syntenic to the distal part of human 21q22.3. It contains 47 genes orthologous to their counterpart on Hsa21 (http://www.ensembl.org). Comparative CGH array analysis on DNA extract from Ms4Yah and wild-type control mice confirmed the loss of one copy of the genes contained within the *Prmt2*–*Cstb* interval and indicated that there was no other chromosomal alteration apart from the expected one (Fig. 1). Tc1 mice were generated by introducing a complete Hsa21 in mouse ES cells using the technique of irradiation microcell mediated chromosome transfer [38]. Sequencing and CGH array analysis of Hsa21 in Tc1 mice revealed that the chromosome is incomplete and bears some rearrangements. In addition to the three deletions already described [Del1 (*C21orf37-PRSS7*), Del2 (*C21orf45-RUNX1*) and Del3 (*COL18A1-PCBP3*)] [45], a duplication of genes *S100B* and *PRMT2* with partial duplication of *DIP2A* was also detected (Fisher and Tybulewicz, personal communication).

A standard breeding strategy was used to generate compound mutant mice carrying the Tc1 transchromosome and Ms4Yah deletion allele together or separately. In order to get the different groups needed for the analysis, 30 Tc1 females were necessary to breed with Ms4Yah males. Tc1, Ms4Yah and Tc1Ms4Yah mice were all present at a normal Mendelian ratio (24% wildtype, 26% Ms4Yah, 31% Tc1, 20% Tc1Ms4Yah for 133 newborns). In addition this result was verified by the segregation of the Tc1 in the F1B6C3B genetic background (47% wildtype and 53% Tc1 out of 196 newborns).

### 4.2 Deficits in motor coordination are confirmed in the Tc1 mice but not rescued by the loss of trisomy in the *Cstb*–*Prmt2* region

**Fig. 2:**
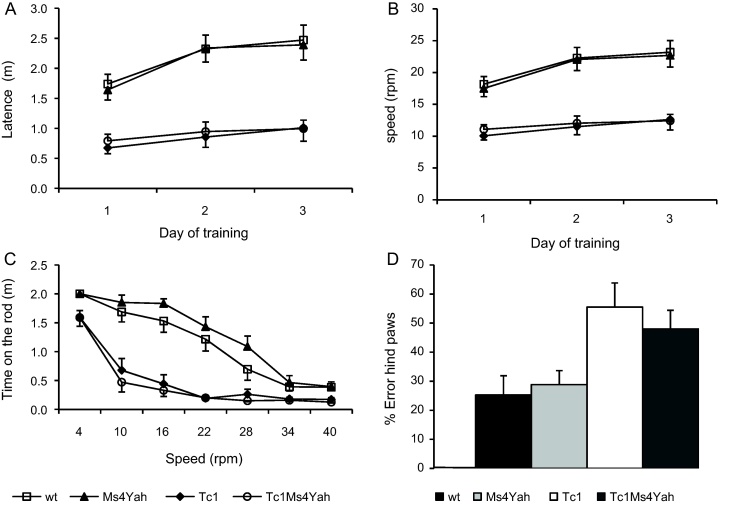
Locomotor performance: training phase on rotarod test. (A) Results are expressed as time (min) that mice remained on an accelerating rota rod (4–40 rpm over 5 min) before falling. (B) Mean rotational velocity at the time of falling. (C) Test phase. The graphs plot the time (min) that mice stayed on the rotarod when tested at constant speeds between 4 and 40 rpm. (D) Notched bar test, results are express in percentage of errors made by the mice with her hind paws when crossing the notched bar. The conclusion from these experiments is that Tc1 mice show impaired motor performance and deletion of *Cstb*–*Prmt2* region does not rescue this phenotype. Values represent means + S.E.M.

Mice were evaluated for their motor skills using the rotarod test. During the training period made of three daily training sessions each one with 4 trials during which the mice were placed on an accelerating rotarod, the latencies to fall off the rod were recorded and a mean latency to fall for the 4 trials was calculated. For the 3 sessions, mice bearing the transchromosomic Hsa21 performed less well than the other genotypes, spending less time on the rod before falling (repeated ANOVA “days”, “genotype” Tukey post hoc analysis; *F*_(3,153)_ = 44.85, *p* < 0.001, wildtype vs Tc1, *q* = 12.019, *p* < 0.001; wildtype vs Tc1Ms4Yah, *q* = 11.44, *p* < 0.001, Ms4Yah vs Tc1, *q* = 11.74, *p* < 0.001, Ms4Yah vs Tc1Ms4Yah, *q* = 11.15, *p* < 0.001; Fig. 2A). Similar result was obtained for the speed at which Tc1 mice fell from the rod, which was slower than those of wt (wildtype) mice (Fig. 2B). Moreover, while wildtype and Ms4Yah mice were able to improve their performance during the training sessions (ANOVA “days”, “genotype”; Tukey post hoc analysis: *F*_(2,153)_ = 7.932, *p* < 0.001, wildtype day3 vs day1, *q* = 3.75, *p* = 0.022; Ms4Yah day3 vs day1, *q* = 3.98, *p* = 0.013), Tc1 and Tc1Ms4Yah mice did not perform better at the end of the training (Tc1 day3 vs day 1, *q* = 1.78, *p* = 0.416; Tc1Ms4Yah day3 vs day 1, *q* = 1.08, *p* = 0.724).

The phase test consisted of two sessions of 6 consecutive trials of 2 min at constant speed, with increasing speed between each trial (4, 10, 16, 22, 28, 34 and 40 rpm). As for the training sessions, a significant difference was observed in performances between Tc1 and Tc1Ms4Yah on one side, and wildtype and Ms4Yah genotypes on the other side at the speeds 10 rpm to 28 rpm (ANOVA “genotype”, “speed”; Tukey post hoc: *F*_(3,357)_ = 82.62, *p* < 0.001). No significant difference in the motor performance between wildtype and Ms4Yah (*q* = 2.934, *p* = 0.161) and between Tc1 and Tc1Ms4Yah (*q* = 1.512, *p* = 0.708; Fig. 2C) were found. All the data reported here confirmed the locomotor phenotypes of the Tc1 mice in this new genetic background but no influence of the disomic rescue for the *Cstb*–*Prmt2* region in the Tc1Ms4Yah compound mice.

We used the notched bar test to investigate hind limb coordination. Only 12 wildtype, 10 Ms4Yah, 11 Tc1 and 11 Tc1Ms4Yah accepted to do the task. Despite the small number of tested mice, significant genotype difference was observed for the percentage of errors in hindlimb coordination. Again, Tc1 and Tc1Ms4Yah mice committed approximately 25% more errors than wildtype and Ms4Yah mice. This was statistically significant for the Tc1 mice (ANOVA “genotype”, Tukey post hoc analysis, *F*_(3,40)_ = 4.76, *p* = 0.006, wildtype vs Tc1, *q* = 5.58 *p* = 0.013, Ms4Yah vs Tc1, *q* = 3.8 *p* = 0.045) whereas Tc1Ms4Yah mice showed a tendency for increased errors (wildtype vs Tc1Ms4Yah, *q* = 3.44 *p* = 0.087; Fig. 2D). Locomotor phenotypes were scored in Tc1 mice as previously described but no contribution of the *Cstb*–*Prmt2* region was detected and the Tc1 and the Tc1Ms4Yah compound mice showed similar defects.

### 4.3 Tc1 and Tc1Ms4Yah mice show specific exploratory behaviour in the open field

**Fig. 3:**
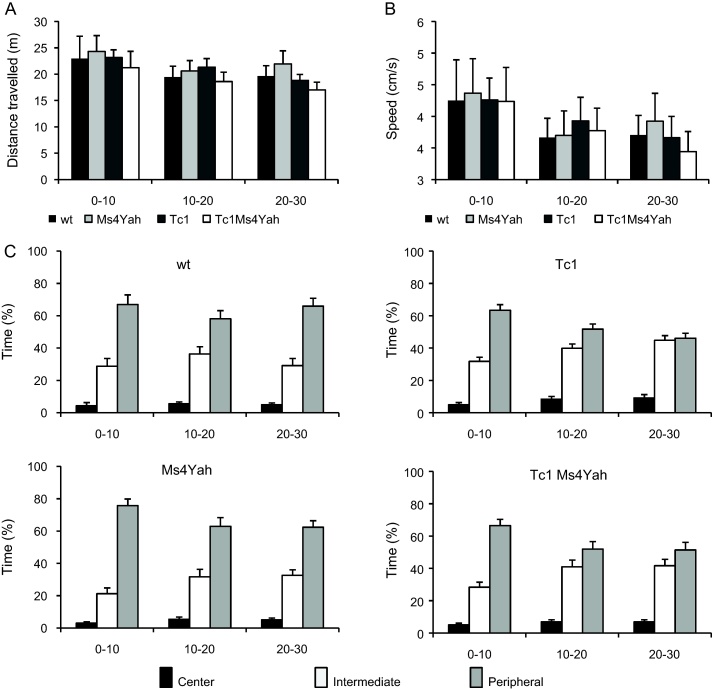
Open field locomotor activity of mice. Locomotion, the results are expressed as distance travelled (m) (A) and the mean speed (m/s) (B) of mice introduced in the open-field test. This data not show any hyperactivity in transchromosomic mice compared with non transchromosomic. (C) Open-field exploration pattern. Mice are scored for the percentage of time spent in the central (black bars), intermediate (white bars) and peripheral (grey bars) areas over 30 min of test, fragmented into 3 sessions of 10 min. This analysis reveals a difference of exploration pattern between Tc1 and Tc1Ms4Yah versus wildtype and Ms4Yah mice. Values represent means + S.E.M.

Locomotor activity and exploratory behaviour were also analysed in the open field during 30 min. Spontaneous horizontal locomotion was measured by recording the distance travelled by the animal and its mean speed during 3× 10 min sessions. No difference was found in the travelled distance (repeated ANOVA “genotype” “session”, *F*_(3,98)_ = 0.443, *p* = 0.723) and velocity (repeated ANOVA “genotype” “session”, *F*_(3,98)_ = 0.0431, *p* = 0.988; Fig. 3A and B) for the four different genotype groups, revealing no hyperactivity of the mice.

Zone analysis revealed different exploration patterns between wildtype and Ms4Yah mice on one side and Tc1 and Tc1Ms4Yah mice on the other side. While the percentage of time spent by wildtype and Ms4Yah mice gradually decreased from the periphery to the centre, the percentage of time spent by Tc1 and Tc1Ms4Yah mice in the periphery ring and intermediate area was identical. This was already visible after 10 min that the mice spent in the arena (repeated ANOVA “genotype”, “zone”, *F*_(2,98)_ = 128.032, *p* < 0.001, Tukey post hoc analysis, wildtype periphery vs intermediate, *q* = 4.866, *p* = 0.002; Ms4Yah periphery vs intermediate, *q* = 6.991, *p* < 0.001; Tc1 periphery vs intermediate, *q* = 2.656, *p* = 0.151; Tc1Ms4Yah periphery vs intermediate, *q* = 2.551, *p* = 0.174). This particular profile was confirmed during the last 10 min (repeated ANOVA “genotype”, “zone”, *F*_(2,98)_ = 149.441, *p* < 0.001, Tukey post hoc method, wildtype periphery vs intermediate, *q* = 8.855, *p* < 0.001; Ms4Yah periphery vs intermediate, *q* = 7.171, *p* < 0.001; Tc1 periphery vs intermediate, *q* = 0.309, *p* = 0.974; Tc1Ms4Yah periphery vs intermediate, *q* = 2.435, *p* = 0.202). Hence, mice bearing the Tc1 chromosome spent less time in the periphery ring (Fig. 3C) but change in the copy number of the *Ctsb*–*Prmt2* region did not affect the exploratory behaviour.

### 4.4 Short-term memory impairment in Tc1 mice is not affected by the deletion of the *Cstb*–*Prmt2* region

**Fig. 4:**
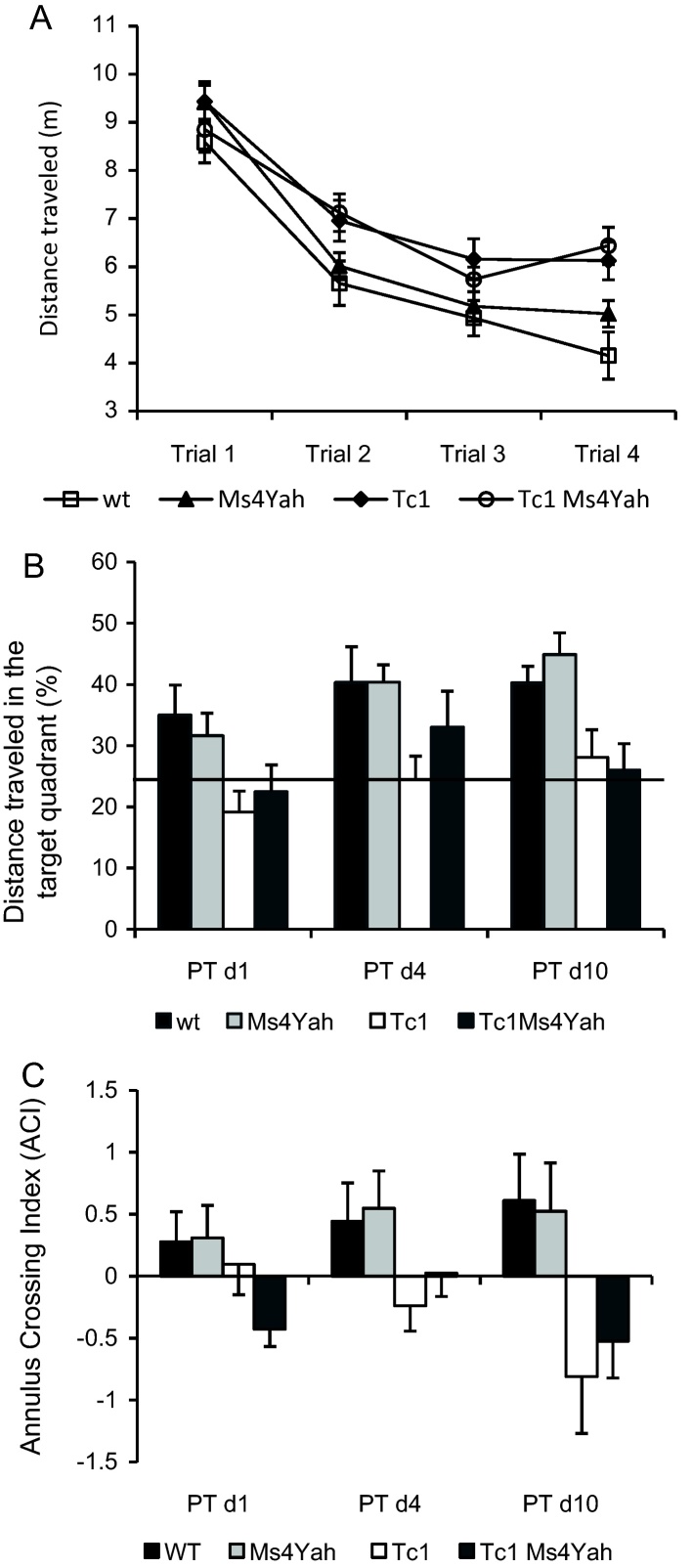
Spatial working memory performance of mice in the Morris watermaze. (A) Acquisition trials, the results are expressed as distance travelled (m) and show increased distance travelled by the Tc1 and Tc1Ms4Yah to reach the PF. (B) Probe trials of the spatial WM version. Mice are scored for the percentage of distance travelled in the target quadrant. The horizontal line indicates the distance travelled using a random search strategy (25%). (C) Annulus crossing index of probe trials. This result confirms the impaired spatial WM in Tc1 and Tc1Ms4Yah mice. Values represent means + SEM.

We further investigated learning and memory in the Morris watermaze, plus we also investigated working memory specifically. Data were analysed separately for each training session, every day being considered as a new experiment. Four trials were administered daily and the distance travelled on each trial was averaged for the 11 days of training. In this experiment, one wildtype animal was removed because of its passive behaviour in the swimming pool. During this spatial hippocampal-dependent learning phase with the hidden platform, the distance travelled to find the platform decreased with training (successive acquisition trials), with a statistically significant reduction between trial 1 (T1) and trial 4 (T4; Fig. 4A; repeated ANOVA “trials”, “genotype”, *F*_(3,150)_ = 104.607, *p* < 0.001, Tukey post hoc method, wildtype T1 vs T4: *q* = 12.574, *p* < 0.001; Ms4Yah T1 vs T4: *q* = 13.478, *p* < 0.001; Tc1 T1 vs T4: *q* = 10.135, *p* < 0.001; Tc1Ms4Yah T1 vs T4: *q* = 9.536, *p* < 0.001) indicating that all the groups of mice were able to learn. But Tc1 mice and Tc1Ms4Yah showed a delay in the acquisition of the task and travelled a greater distance to find the platform than wildtype and Ms4Yah mice in each trial, with a difference in performance between the two groups increasing with the acquisition trials and being clearly significant at trial 4 (repeated ANOVA “trials”, “genotype”, *F*_(3,150)_ = 5.7, *p* = 0.002, Tukey post hoc method: wildtype vs Tc1: *q* = 5.039, *p* = 0.002; wildtype vs Tc1Ms4Yah: *q* = 5.833, *p* < 0.001).

During probe trials, retention tests indicated a marked preference of the wildtype and Ms4Yah mice for the targeted quadrant (TQ) versus non targeted quadrant (NTQ), whereas Tc1 and Tc1/Ms4Yah mice performed at close to chance level (25%), indicating a defect in hippocampal short-term retention memory (Fig. 4B; probe test day 10: ANOVA “genotype”, “quadrant”, *F*_(3,100)_ = 9.027, *p* < 0.001, Tukey post hoc analysis, wildtype TQ vs NTQ: *q* = 6.590, *p* < 0.001; Ms4Yah TQ vs NTQ: *q* = 9.272, *p* < 0.001; Tc1 TQ vs NTQ: *q* = 1.452, *p* = 0.307; Tc1Ms4Yah TQ vs NTQ: *q* = 0.472, *p* = 0.739). Probe test analysis by computing an annulus crossing index (ACI) (see Section 2) revealed that, contrary to the wildtype and Ms4Yah mice that had a positive ACI, mice bearing the Tc1 chromosome had a negative ACI for the third probe test. They crossed the platform position less frequently, indicating that they had not established any spatial strategy (Fig. 4C; ANOVA “ACI”, “genotypes”, *F*_(3,100)_ = 5.820, *p* = 0.002, Tukey post hoc analysis, wildtype vs Tc1: *q* = 4.685, *p* = 0.005; Ms4Yah vs Tc1: *q* = 4.577, *p* = 0.007; wildtype vs Tc1Ms4Yah: *q* = 3.743, *p* = 0.041; Ms4Yah vs Tc1Ms4Yah: *q* = 3.596, *p* = 0.054). Search paths from sessions of days 6 to 11 were further classified depending on the type of strategy and revealed that Tc1 and Tc1Ms4Yah mice used more random search strategy than wildtype and Ms4Yah mice in all four trials (Table 1). This result being stress-dependent, we controlled thigmotaxis behavior during the experiment and found no increased time spent at the periphery of the field (repeated ANOVA “trials”, “genotypes”, *F*_(3,150)_ = 1.007, *p* = 0.397). A cued version was performed after the series of spatial-learning-reversals test on the first group of mice. The distance travelled to reach the visible platform was similar in all the groups of mice (repeated ANOVA “genotype”, trials”, *F*_(3,147)_ = 0.157, *p* = 0.925). As previously no changes in the Tc1 phenotypes, with or without the Ms4Yah, were observed in the different tests.

### 4.5 Tc1 and Tc1Ms4Yah mice exhibit impaired social preference

Thus we decided to go further to test additional paradigms for the Tc1 Hsa21 in order to evaluate the contribution of the *Cstb*–*Prmt2* genetic interval. First we looked at social preference. During the habituation phase of the test, mice had no preference for one or the other empty test chamber (*F*_(1,98)_ = 1.092, *p* = 0.299). They spent the same time visiting each chamber, indicating that there was no bias in the sociability test due to place preference. In addition, there was no effect of genotype on time spent visiting each room (*F*_(3,98)_ = 0.303, *p* = 0.823) (data not shown). During this phase, one wildtype mouse and one Tc1 mouse were removed from the test because they did not visit either of the chambers.

**Fig. 5:**
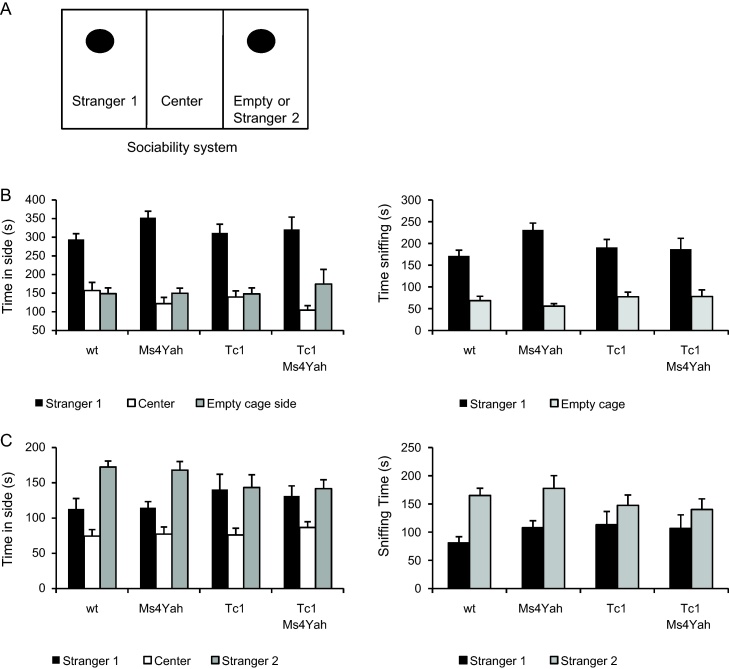
Sociability and novelty performance of the mice. (A) Representation of the apparatus for social behaviour test. The system is composed of three successive identical chambers with openings allowing access between the chambers. The black circle indicates the position of the wire cage containing the stranger1 and empty wire for the sociability test or the two strangers for the novelty test. (B) Time spent in each side (left panel) and time spent sniffing each wire cage (right panel) during the tests for sociability. All mice demonstrated a strong preference for the wire cage containing an unfamiliar mouse (stranger 1). (C) Time spent in each side (left panel) and time spent sniffing each wire cage (right panel) during the tests for novelty. Whereas wildtype and Ms4Yah mice showed a significant preference for the wire cage containing the second unfamiliar mouse (stranger 2), Tc1 and Tc1Ms4ayh mice spent equal time with the stranger 1 as with stranger 2. Data shown are mean + S.E.M. for each strain.

During the first part of the test, investigating sociability of the mice, all the mice whatever their genotype displayed equivalent significant preference for spending time in the chamber with the first stranger mouse (ANOVA “genotype”, “time within chambers”, *F*_(1,98)_ = 94.477, *p* < 0.001) (Fig. 5B, left panel). Likewise, the measure for the time spent sniffing at the wire cage containing the stimulus mouse versus the empty wire cage also indicated a strong preference of all groups of mice for the cage containing the other mouse (*F*_(1,98)_ = 129.023, *p* < 0.001) (Fig. 5B, right panel). This measure is a good indication of more direct social interest.

During the second part of the test, the preference for social novelty is measured by placing a new stranger mouse in the empty wire cage and comparing the interactions of the test mice with the familiar stranger 1 mouse and the novel stranger 2 mouse. As for the sociability test, there was a significant effect on preference for the newly introduced stranger 2 (*F*_(1,98)_ = 9.558, *p* = 0.003). However, while wildtype and Ms4Yah mice demonstrated a significant preference for stranger 2 (Tukey post hoc: wildtype, stranger1 vs stranger2, *q* = 3.929, *p* = 0.007; Ms4Yah, stranger1 vs stranger2, *q* = 3.8005, *p* = 0.008), Tc1 and Tc1Ms4Yah mice spent as much time with the stranger 1 as with stranger 2 (Tukey post hoc: Tc1, stranger1 vs stranger2, *q* = 0.207, *p* = 0.884; Tc1Ms4Yah, stranger1 vs stranger2, *q* = 0.743, *p* = 0.601) (Fig. 5C, left panel). The same results are found for the time spent sniffing the stranger mice (repeated ANOVA “genotype”, “time in chambers”, *F*_(1,98)_ = 16.575, *p* < 0.001, Tukey post hoc, wildtype, stranger1 vs stranger2, *q* = 4.173, *p* = 0.010; Ms4Yah, stranger1 vs stranger2, *q* = 3.745, *p* < 0.001; Tc1, stranger1 vs stranger2, *q* = 1.765, *p* = 0.215; Tc1Ms4Yah, stranger1 vs stranger2, *q* = 1.776, *p* = 0.212) (Fig. 5C, right panel). Hence, whereas Tc1 and Tc1Ms4Yah mice displayed no deficit in sociability compared with wild-type and Ms4Yah mice during the first part of the test, they did not display a preference for the novel mouse during the second part, thereby suggesting a deficit in social preference.

### 4.6 Olfaction is not impaired in the different genotypes

**Fig. 6:**
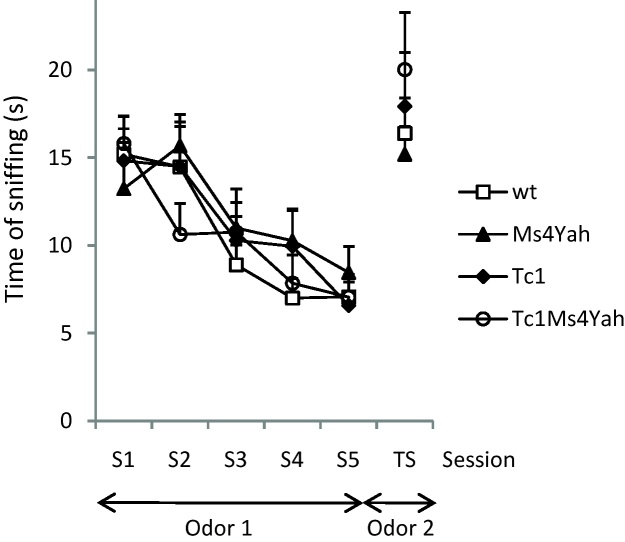
Olfactory test expressed in time spent sniffing olfactory stimuli in repeated presentations (S1–S5). All mice showed change in the amount of time spent sniffing the new olfactory stimuli in the test session. Data shown are mean + S.E.M. for each strain.

As a control experiment we also tested the olfactory system of the mutant mice. All the mice showed a clear change in behaviour when confronted with the new olfactory stimulus at the test session (TS), or after five sessions of habituation (S5) (Fig. 6; repeated ANOVA “genotype”, “session”, *F*_(3,180)_ = 22.738, *p* < 0.001, Tukey post hoc, wildtype S5 vs TS, *q* *=* 5.708, *p* < 0.001; Ms4Yah S5 vs TS, *q* = 4.128, *p* = 0.041; Tc1 S5 vs TS, *q* = 6.957, *p* < 0.001; Tc1Ms4Yah S5 vs TS, *q* = 7.949, *p* < 0.001). Overall no significant effect of genotype was observed for the time spent sniffing the two olfactory stimuli (ANOVA “genotype”, “session”, *F*_(3,180)_ = 0.118, *p* = 0.949). This indicates that all the genotypes had similar ability to discriminate between unfamiliar and familiar olfactory stimuli. Hence, there is no impact by the transchromosome or the deletion on the smelling capacity of the mice.

## 5 Discussion

### 5.1 Transmission of genetic modifications

Ms4Yah mice monosomic for the *Prmt2*–*Cstb* region on Mmu10 were crossed with the Down syndrome mouse model Tc1, trisomic for ∼81% of the Hsa21 genes, to test the role of the 47 mouse genes with homologues in human present in the *Prmt2*–*Cstb* region in the phenotypes observed in Tc1 mice.

Ms4Yah mice were obtained through *in vivo* chromosomal engineering using a mouse line containing *loxP* sites in a cis configuration and the *Prmt2* and *Cstb* loci crossed with a general CRE deleter strain, *Tg*(*CMVCRE*)*1pcn*. Successive crosses of this monosomic line always resulted in the birth of Ms4Yah pups at a Mendelian ratio, indicating no lethality of the *Prmt2*–*Cstb* interval when present in only one copy. Unlike previous reports [38] the Hsa21 in the F1B6C3B Tc1 line was transmitted in a normal Mendelian manner. This suggests an absence of lethal cardiac anomalies of the Tc1 pups in our B6C3B genetic background as it is possible that cardiac defects may account for the loss of Tc1 compared to wildtype littermate pups by weaning. It is not surprising as the presence of such anomalies has been shown to be dependent on the genetic background in Tc1 mice. Dunlevy et al. [15] have shown increased incidence of cardiac malformations in Tc1 mice on pure C57BL/6J background than on mixed 129S8:C57BL/6J (F1). Hence, we can hypothesize that there is a compensatory effect coming from the mixed genetic background used in the present study. Consequently the mapping of the euploid modifier genes which are involved in such genetic interaction could be envisaged.

### 5.2 Confirmation of locomotor and short-term memory impairments in Tc1 mice with no contribution of the *Cstb*–*Prmt2* region for these phenotypes

Tc1 mice have already been extensively explored for phenotype relationships to DS [38,34,19]. In particular, Tc1 mice have been analysed in the rotarod, open field and Morris water maze tests that revealed they had impaired motor coordination, hyperactivity and short-term memory deficits [34,19]. Hence Tc1 animals provide a good internal control for our analyses. Indeed, during our investigation, we could recapitulate all these phenotypes, except one, in our B6C3B Tc1 mouse colony.

Tc1 mice have been previously shown to have severe deficits in motor skills in different motor coordination tasks such as the rotarod, static rod and footprint tests [19]. Rotarod performance analysis in our study confirmed the deficit in locomotor activity of the Tc1 mice. We also used a test more specific for balance and movement coordination. The notched bar-crossing test investigates hindlimb motor coordination by measuring the number of hindpaw slips when walking on a notched bar. This test confirmed the impairment of motor coordination of the Tc1 mice.

In the open field, Tc1 mice spent less time at the periphery and had decreased thigmotaxic behaviour. The same behaviour was observed by Galante et al. [19]. In this first analysis, this behaviour was attributed to the hyperactivity of the mice. The hyperactive phenotype in Tc1 mice seemed robust as it has been observed in two different studies, with two different genetic backgrounds (C57BL/6 × 129S8 F2 and C3H/HeJ) and in two different paradigms (open field and Morris watermaze [38,34,19]). However, we did not observe any hyperactivity of the Tc1 mice in our different tests, be it in the open field, in the Morris watermaze (data not shown) or in the social recognition test. Previous behavioural studies on Tc1 mice were done on B6129S8 background [34,19]. Even if the gene regulation of this trans-chromosome seems to be to determine by the human sequence, small exceptions are present [7]. Thus the implication of modifiers murine genes on the human sequences of the transchromosome can alter phenotype, as suggested previously [10]. In this study, we used a F1CB6C3B background derived from the C3H/HeSnJ (C3H) mouse strain in which the mutated *Pde6b* allele normally present on this background was replaced by a functional allele from BALB/c [20]. Similar F1B6C3B background was already used to validate DS-like phenotypes in the Ts65Dn model [9]. Difference in the exploration activity in this background could depend on several genes as many differences exist between C3H and 129 backgrounds. Nevertheless we consider *Disrupted in schizophrenia 1* (*Disc-1*), as a candidate. Indeed *Disc-1* is mutated in 129 strains compared to C3H and found identified as genetic risk factor in schizophrenia and affective disorders [6], inducing deficit in working memory [25,27]. Furthermore inactivating *Disc-1* in mice induced hyperactivity and an impact on neuronal organization, number of synapses, hippocampal plasticity and on the proliferation of neural progenitor cells [31]. In addition Pericentrin 2 encoded by *Pcnt2* found in the *Ms4Yah* deleted interval has been associated with schizophrenia and bipolar disorder by several studies and is known to interact with *Disc-1* [1,2]. Of course additional genetic regions of 129P2 could control the low level of activity of knockout strains as illustrated by the following studies [16,17].

Tc1 mice have previously been shown to have impaired spatial working memory [34]. However, presence of the deleted *Disc-1* gene in this study again interfere with the study as it has been shown that mice that are either heterozygous or homozygous for the defective *Disc-1* gene have an altered working memory in an alternation test [25]. The impact of absence of the *Disc-1* protein in the context of the Tc1 mosaicism is difficult to assess due to the complexity and variability of Tc1 phenotypes. Testing spatial working memory with a series of platform re-positionings in the Morris watermaze with Tc1 mice free of the *Disc-1* mutation confirmed the deficit of spatial working memory due to the transchromosome.

Looking more precisely to the swimming tracks, it appears that Tc1 mice use more random strategy and do not set up efficient spatial search strategy compared to wildtype littermates. However, the method used here to classify strategies into unique categories generates a bias because it does not take into account combined strategies or the chance factor. Nevertheless, this method reflects dominant strategies used by the animals during the last 6 days of the test [23]. The observed phenotype is also robust enough to compensate for the mosaicism of the Tc1 mice. Tc1 mice display a high degree of mosaicism as the Hsa21 is lost from a subset of cells, with a degree varying between tissues. This mosaicism might be at the origin of the heterogeneity of the phenotypes observed in Tc1 mice. This assumption is supported by studies conducted in human showing a correlation between the level of mosaicism and the severity of the phenotype [41].

Mice were tested for social interaction. In the first part of the test investigating the sociability of the mice, all the mice whatever their genotype have a normal behaviour. Indeed, the mouse is a social animal and tends to prefer to stay in the company of another mouse rather than in an empty place. However, during the second test phase investigating the preference for social novelty, the mice bearing the Hsa21 chromosome did not show any preference for the new mice introduced compared to the old one. Studies of inbred mouse strains have shown that the sociability test and the preference for social novelty test involve separate systems [36,37]. Social recognition in the mouse is mainly based on the olfaction. When a mouse encounters another individual, it smells its head and urogenital area for about 1 min to collect olfactory information. If encountering the same mouse again, it will smell it only a few seconds to remember it and will then engage in other behaviours. This social memory was shown to be independent of the elapsed time between two presentations [11]. Hence, Tc1 mice seem to have a defective social memory. This phenotype was until now never described in Tc1 mice or in other DS models. Social memory has been shown to be sustained by the amygdala [54], but also by the hippocampus [47]. Hence, many of the phenotypes that we are describing here involve the hippocampus and the deficits associated with the “Tc1” genotype.

### 5.3 Absence of phenotype associated with the monosomy of the *Cstb*–*Prmt2* region

For all the tests realized here, we did not observe any deficit in the Ms4Yah mice. The Ms4Yah allele corresponds to a deletion of the *Cstb*–*Prmt2* region of Mmu10. Hence, Ms4Yah mice are monosomic for this region corresponding to the telomeric part of Hsa21. Both syndromes are due to gene dosage errors that disturb a variety of physiological and morphological systems. Contrary to DS, monosomy 21 is a rare human disease, with only cases of partial monosomy or mosaicism surviving after birth. Signs and symptoms of monosomy 21 are broadly similar to those of Down syndrome and are very variable in surviving patients and depend on the piece of the Hsa21 that is haploinsufficient. Still, in the majority of partial monosomies 21, mental retardation is the major clinical manifestation.

The absence of behavioural phenotype linked to the monosomy of the *Cstb*–*Prmt2* region in Ms4Yah mice observed in this study confirms studies realized on human patients with partial trisomy 21 having a deletion of the same region and who presented phenotypes similar to DS [40]. This is further supported by the discovery of 5 cases of partial monosomy of the distal part of Hsa21 with deletion of the S100B gene but with no associated phenotypes [32]. Our previous work showed that a smaller deletion from *Col6A1* to *Prmt2* displayed no behaviour phenotype using similar paradigm but we found additional lung and inflammatory phenotypes [4]. In addition, two cases of patients having ring chromosome 21 associated with a loss of the telomeric part of Hsa21 (from *D21S112* to *S100b*) did not present any abnormal phenotype. Yu and collaborators published a paper in which they described the generation and behavioural analysis of new mouse models of monosomy 21. One of these models corresponds to the engineered deletion of the *Prmt2–Pdxk* region. The *Pdxk* gene is present 3′ downstream of *Cstb* on Mmu10 and hence the Df(10)1Yey model is larger than our Ms4Yah model. These models were analysed in the open field, Morris watermaze, contextual and fear conditioning tests [55]. Df(10)1Yey mice showed impairment in spatial and learning reference memory as assessed by the Morris watermaze test and associative memory as assessed by fear conditioning test. These cognitive phenotypes were not tested in the present study as we based our investigation on the spatial reference memory that has been described to be defective in Tc1 mice. These new phenotypes indicate that the *Cstb*–*Prmt2* region has some implication in the Monosomy 21 pathology and that more analyses have to be carried out to reveal more phenotypes.

### 5.4 Tc1 phenotypes are not rescued by the euploidy of the *Cstb*–*Prmt2* region

By mating Ms4Yah mice with Tc1 mice, we could investigate the role of the *Cstb*–*Prmt2* region within the Tc1 context. In all the tests where phenotypic differences appeared, it was possible to separate mice groups depending on the presence or not of the Hsa21 chromosome. When the Tc1 group of mice presented deficits, the Tc1Ms4Yah mice presented the same deficit and hence there was no rescue of the Tc1 deficit by coming back to diploid of the *Cstb*–*Prmt2* region. Until now, neither partial trisomic patient for the *Cstb*–*Prmt2* region, nor mouse model trisomic only for this region has been described. Hence, there is no direct evidence of the contribution of genes present in this region to the Down syndrome phenotype. The strategy that we have used is hence of particular interest to help to decipher the contribution of this region to DS [26].

One has however to take into account the presence of deletions on the Hsa21 present in Tc1 mice, resulting in the presence in only copies of the *Slc19a1*, *Col18a1* and *Pcbp3* genes [45]. Tc1 mice are not trisomic for those genes whereas Tc1Ms4Yah mice are monosomic for them. The role of these genes cannot therefore be investigated here. Hence, the motor deficits observed in a mouse model over expressing *Slc19a1* could not be assigned to the *Cstb*–*Prmt2* region [22]. In addition to the loss of some telomeric genes in the Hsa21 transchromosome, other modifications have also been described, in particular two other deletions between the *Cxadr* and *D21S1922* (3.4 Mb) markers and between *Ifnar1* and *Runx1* (1.5 Mb) [38]. Hence, the *Cstb*–*Prmt2* region could have a role in DS pathologies either not present in the Tc1 model or in collaboration with genes that are not triplicated in the Tc1 model.

One has also de keep in mind that experiments that were undertaken here have been pursued with mice with an F2 hybrid genetic background coming from an F1 B6C3B cross. The genetic heterogeneity due to crossing-over in F1 gametes can potentially result in offspring with different genetic backgrounds and hence in a population of animals genetically more heterogeneous than a population coming from a pure breeding. This heterogeneity could have an effect that will mask that of the *Cstb*–*Prmt2* region if this one is weak.

The increasing number of DS mouse models and their analysis has enabled us to highlight the complexity of the genetic interactions that underlie the pathology associated to DS. Hence, the Ts1Yah model trisomic for the *Abcg1-U2af1* region alone has an increased LTP [43], whereas another mouse model trisomic for all Hsa21 syntenic regions has a diminished LTP [56]. The same apparent contradictory phenotypes were found in with the craniofacial analysis of mouse models for different segments of the Mmu16 homologous region [39], suggesting that several genes or regions might have negative or positive contributions with different effect depending on their genetic environment. These findings demonstrate the relevance of this “rescue” investigation. This study will hence be pursued with the analysis of a new mouse model trisomic for the *Cstb*–*Prmt2* region. In addition, additional rescue experiment with other monosomic model and Tc1 mice are valuable to decipher the contribution of other regions to the Tc1 phenotypes. Conversely, an additive approach by mating partial trisomic models or partial monosomic models together for the telomeric part of the Hsa21 will enable us to decipher the genetic mechanisms involved in the development of DS pathologies.

## Figures and Tables

**Table 1:** Percentage of random strategy of the different mice during the acquisition trial of the working memory test in the Morris water maze.

	Percentage of Random Strategy			
	Trial 1	Trial 2	Trial 3	Trial 4
Wildtype	20.83	15.28	12.50	13.89
Ms4Yah	34.52	15.48	10.71	15.48
Tc1	45.24	36.90	35.71	35.71
Tc1Ms4Yah	48.81	45.24	33.33	38.10
